# Broadband Asymmetric Light Transmission at Metal/Dielectric Composite Grating

**DOI:** 10.1038/s41598-018-19329-7

**Published:** 2018-01-17

**Authors:** Rui Zhu, Xuannan Wu, Yidong Hou, Gaige Zheng, Jianhua Zhu, Fuhua Gao

**Affiliations:** 1 School of Physical Science and Technology, Sichuan University, Chengdu, Sichuan 610064 China; 2 Jiangsu Key Laboratory for Optoelectronic Detection of Atmosphere and Ocean, School of Physics and Optoelectronic Engineering, Nanjing University of Information Science and Technology, Nanjing, 210044 China

## Abstract

Optical diode-like effect has sparked growing interest in recent years due to its potential applications in integrated photonic systems. In this paper, we propose and numerically demonstrate a new type of easy-processing metal/dielectric cylinder composite grating on semi-sphere substrate, which can achieve high-contrast asymmetric transmission of unpolarized light for the sum of all diffraction modes in the entire visible region, and effectively guide the diffraction light transmitting out the substrate. The asymmetric light transmission (ALT) ratio is larger than 2 dB in the waveband from 380 nm to 780 nm and the maximum ALT ratio can reach to 13 dB at specified wavelengths. The thorough theoretical research reveals that the proposed metal/dielectric pillar composite grating structure, together with the substrate, can effectively excite localized surface plasmonic resonance (LSPR) effect and waveguide mode (WGM), and enlarge the diffraction difference between forward and backward transmission spaces, including both number of diffraction orders and diffraction efficiency, thus resulting in high-contrast broadband ALT phenomenon. In particular, lowering the symmetry of the grating can achieve polarization-dependent ALT. Such a type of easy-processing ALT device with high performance for both polarized and unpolarized light can be regarded as suitable candidates in practical applications.

## Introduction

Optical diode-like effect, referring to the big light transmittance difference between the forward and backward illumination on devices, has attracted tremendous research interests due to its significant potential applications in developing the next generation of all-optical computing and processing devices and systems^1–5^. Typical schemes to achieve asymmetric light transmission (ALT) devices are based on optical nonreciprocity methods, such as magneto-optical effect^2,6,7^, optical nonlinearity^8–10^, indirect inter-band photonic transitions^3,11^ and optoacoustic effect^12^. Optical nonreciprocity is an ideal solution as it enables devices to transmit any optical modes in one direction and block them in the other. However, it is inherently difficult to be realized because it is based on breaking Lorentz symmetry, which is widely existed in the light-matter interaction process^13^. And most of the current optical nonreciprocity methods are usually not compatible with complementary metal-oxide semiconductor (CMOS) process, encouraging researchers to try tremendous solutions to achieve ALT devices.

Recently, reciprocity scheme has been demonstrated to be the other effective method to achieve ALT. Through breaking the spatial symmetry of device, a specific asymmetric mode conversion can be excited, and result in light transmission difference between forward and backward directions. This scheme tactfully bypasses the Lorentz restricted conditions^14^. Comparing with optical nonreciprocity devices, reciprocity devices can not only be realized without limitation to certain materials but also be compatible with CMOS fabrication process. To date, various types of reciprocal ALT devices have been demonstrated based on composite grating structures^15–18^, photonic crystals^19–25^, metal-silicon waveguides^4^, chiral metamaterials^26–29^, hyperbolic metamaterials^30^, and metasurfaces^31–33^. The main issues involved in these devices are diffraction mode (include zero- and high-order diffraction)^16–18^, polarization states of light (include linear polarization and circular polarization state)^26–28^, and the spatial mode (i.e. even and odd modes)^34,35^. These ALT devices can meet more practical application requirements.

Although the reciprocity scheme simplifies the realization condition of ALT devices, the broadband ALT is still challengeable due to material dispersion. Especially for the visible waveband, the structure size which is comparable with ~λ, is still difficult for fabrication under the current state of the art. Recently, Bin Tang suggested a simple ALT device with tapered metallic grating to achieve broadband ALT in visible region^36^. However, the reported ALT device is polarization dependent and it is difficult to control the tapered angle of grating accurately in fabrication process. In this work, we propose and numerically demonstrate a high-performance reciprocity ALT device relied on higher order asymmetric diffraction modes to address all above-mentioned issues. Our device is composed of a type easy-processing metal/dielectric cylinder composite grating (MDCG). The ALT ratio is larger than 2 dB in the entire visible region and the maximum ALT ratio can reach to 13 dB at certain wavelengths. The polarization property is closely related to the cross-profile shape of metal/dielectric pillar: high-symmetric shape (e.g. circle) results in un-polarized ALT effect, while low-symmetric shape (e.g. chiral shape) leads to polarized ALT effect. The diffraction effect, localized surface plasmonic resonance (LSPR) and weak WGM, which are responsible for the high contrast-ratio broadband ALT, have been systematically investigated.

## Schematic and Optimized Results

**Figure 1:**
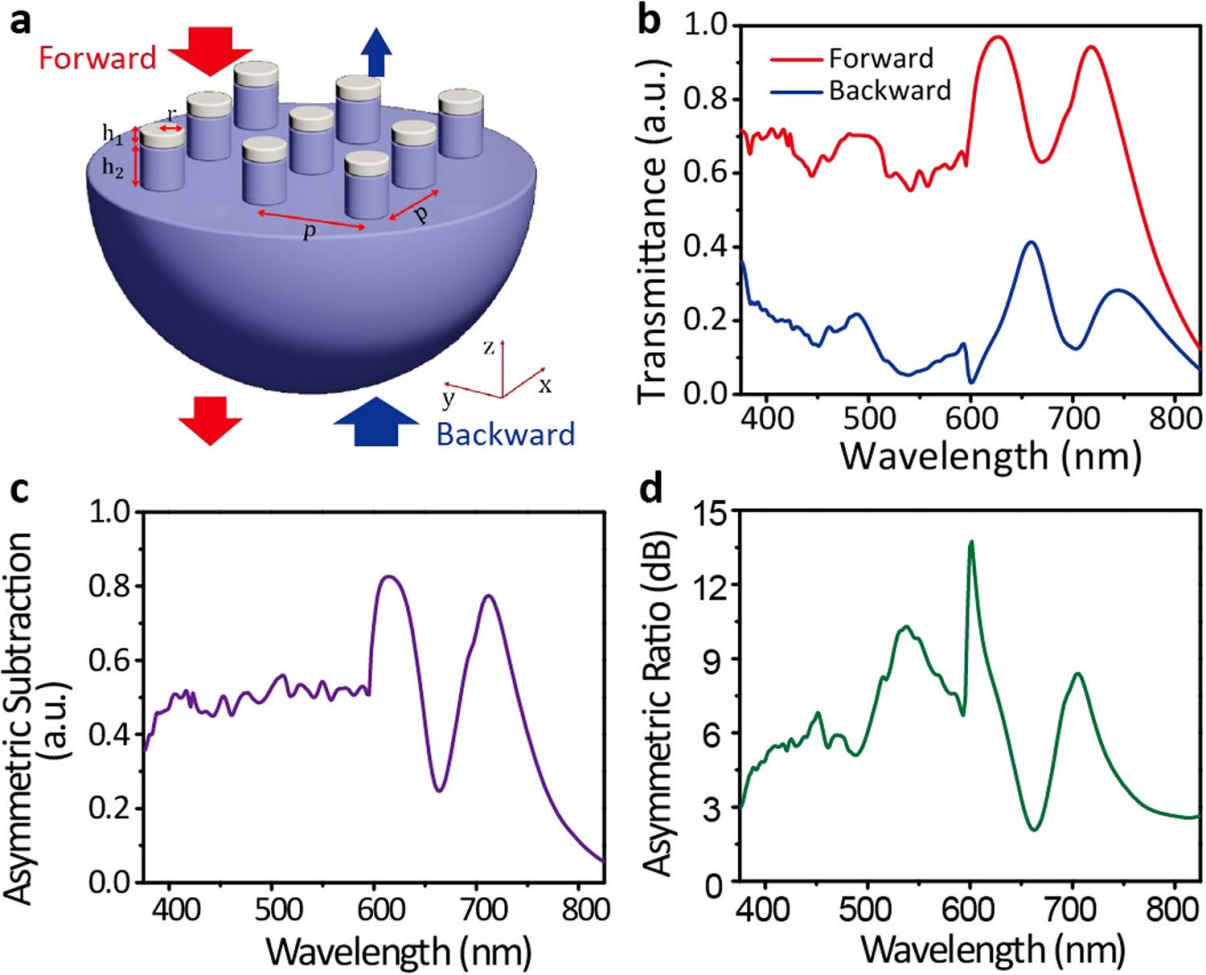
(**a**) Schematic diagram of the designed MDCG. The Ag/dielectric cylinder with radius of *r* is placed periodically on dielectric substrate. The lattice size in both of X- and Y-direction is *P*, and the height of Ag− and dielectric cylinders is *h*_1_ and *h*_2,_ respectively. The dielectric cylinder and substrate take the same refractive index *n* in this work. The red and blue arrows denote the forward (−Z) and backward (+Z) illumination direction, and their width denotes the intensity of the incidence and transmission lights; (**b**) Simulated transmittance spectra under forward (red curve) and backward (blue curve) illumination. The asymmetric subtraction and ratio calculated by these transmittance spectra are shown in (**c**) and (**d**), respectively. The detail structural parameters take the follow optimized values: *h*_1_ = 75 nm, *h*_2_ = 480 nm, *r* = 180 nm, *P* = 600 nm, and ${n}_{l}$ = 1.59.

The schematic of designed MDCG for broadband ALT is shown in Fig. 1(a). The metal/dielectric cylinders are placed periodically on the dielectric substrate. The lattice size in both X- and Y-direction is set as *P* to achieve unpolarized ALT effect. The height of metal and dielectric cylinders is denoted by *h*_1_ and *h*_2_, respectively. To simplify the fabrication process, radius *r* of metal cylinders takes the same value with that of dielectric cylinders, while the refractive index ${n}_{l}$ of dielectric cylinders takes the same value with that of dielectric substrate. Silver (Ag) is used as the metal in our simulation. Indeed, other metals, such as gold (Au) and aluminum (Al), also can be used here, which will result in a similar ALT effect. The forward and backward illumination is defined as light transmitting along the Z− and Z+ direction respectively, as denoted by the red and blue arrows in Fig. 1(a).

To analyze the ALT effect in MDCG, we perform a systematic numerical investigation on the forward and backward transmission properties based on finite-difference time-domain (FDTD) method. The detail information in simulated process can be got in Method Section. Figure 1(b) shows the optimum solution. The forward transmittance is larger than 0.55 in the wave range from 380 nm to 750 nm, while the backward transmittance is smaller than 0.4, indicating the broadband ALT effect in MDCG. It’s worth noting that the maximum forward transmittance can reach to 0.97 at 626 nm, while the corresponding backward transmittance is only about 0.17. Due to the high symmetry of the cylinder and tetragonal lattice, our designed MDCG is insensitive to the polarization states of incident light, resulting in an unpolarized ALT effect. We can expect that polarization dependent ALT effect can be achieved by lowering the symmetry of the pillar or the lattice of MDCG.

To show the asymmetric transmission efficiency more intuitively, we define an asymmetric subtraction and ratio between forward and backward transmittance as

1 $${\rm{Asymmetric}}\,{\rm{Subtraction}}={T}_{{\rm{forward}}}-{T}_{{\rm{backward}}}$$

2 $${\rm{Asymmetric}}\,{\rm{Ratio}}=10\ast \mathrm{log}({T}_{{\rm{forward}}}/{T}_{{\rm{backward}}})$$

where ${T}_{{\rm{forward}}}$ and ${T}_{{\rm{backward}}}$ refer to the forward and backward transmittance respectively. The caculated asymmetric subtraction and ratio based on Fig. 1(b) are shown in Fig. 1(c) and (d), which can reach to 0.5 and 4 dB in the entire visible band respectively, except for the small dip exited at around 660 nm. Especially, the maximum asymmetric subtraction and ratio can reach to 0.8 and 13 dB, indicating our designed MDCG exhibits high asymmetric performance. Through comprehensive analysis, we find that this excellent ALT effect is originated from specific diffraction effect, LSPR and WGM excited in MDCG, which will be systematically discussed later.

## Discussions

### Diffraction effect in MDCG

Diffraction effect is one of the basic physical effects for grating. To gain deep insight into the underlying mechanism for the high-contrast broadband ALT in MDCG, we calculate and analyze the diffraction modes. According to the vectoral diffraction theory^37^, the azimuth angle ${\theta }_{mn}$ and polar angle ${\varphi }_{mn}$ for the specific diffraction order (*m*, *n*) in two-dimensional grating under vertical illumination can be calculated by

3 $${\theta }_{mn}=\arcsin (\frac{\lambda }{{n}_{l}})\sqrt{{(\frac{m}{dx})}^{2}+{(\frac{n}{dy})}^{2}}$$

4 $${\varphi }_{mn}=arctan\frac{n{d}_{x}}{m{d}_{y}}$$

where, $\lambda$ is the wavelength of incident light, ${n}_{l}$ is the background refractive index in diffraction space, $dx$ and $dy$ are the grating periods in X and Y direction. To make the equation have practical physical meaning, ${\theta }_{mn}$ should be limited in the range from 0 to 90 deg. If ${\theta }_{mn}$ is bigger than 90 deg, the corresponding diffraction mode (m, n) cannot transmit through the interface and be blocked in the reflection space. When ${\theta }_{mn}=90\,{\rm{\deg }}$, we can get a critical wavelength ${\lambda }_{c}$ for diffraction mode (m, n)

5 $${\lambda }_{c}={n}_{l}/\sqrt{{(\frac{m}{dx})}^{2}+{(\frac{n}{dy})}^{2}}$$

In our work, $dx=dy=P$. Thus, we have

6 $${\lambda }_{c}={n}_{l}\ast P/\sqrt{{m}^{2}+{n}^{2}}$$

**Figure 2:**
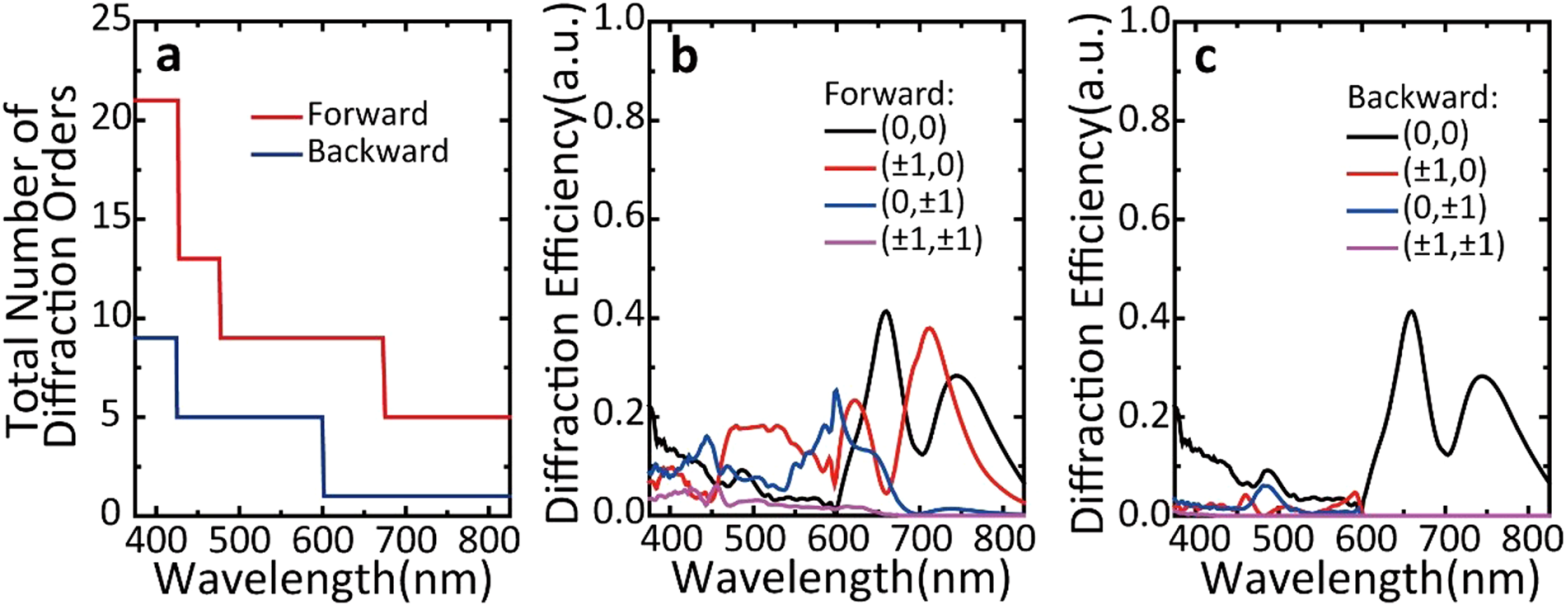
Diffraction effect in MDCG. (**a**) Total number of diffraction orders as a function of wavelength in transmission space under forward (black curve) and backward (red curve) illumination; (**b**) and (**c**) Diffraction efficiency at different diffraction orders under forward and back illumination respectively. The structural parameters used here are the same with that in Fig. 1, where *h*_1_ = 75 nm, *h*_2_ = 480 nm, *r* = 180 nm, *P* = 600 nm, and ${n}_{l}$ = 1.59.

According to Eq. (6), we can easily find that for certain wavelength and period, the number of diffraction modes is determined. Considering when P equals 600 nm, for backward illumination, ${n}_{l}$ equals to 1 which is the refractive index of air while for forward illumination, ${n}_{l}$ is 1.59 which is the refractive index of dielectric substrate. So, we can easily find that the total number of diffraction orders for forward illumination is larger than that for backward illumination for all the wavelengths which is compatible with simulation results shown in Fig. 2(a). Meanwhile, for certain mode (m, n), ${\lambda }_{c}$ is also different for forward and backward illumination. These differences imply the existence of ALT effect.

Figure 2 shows the simulated diffraction result of an optimized MDCG which takes the same parameters as Fig. 1. The total number of diffraction orders for forward illumination is obviously larger than that for backward illumination as shown in Fig. 2(a), which is caused by the refractive index difference in two different transmission spaces. In fact, as indicated by Eq. (6), any one- or two-dimensional grating with different refractive index on two sides may result in ALT effect. What’s more, the diffraction efficiency needs to be considered. Figure 2(b) and (c) shows the diffraction efficiency at different orders under forward and backward illumination, where only lower diffraction orders with high diffraction efficiency are included while higher diffraction orders with low efficiency have been excluded. Comparing Fig. 2(b) and (c), we find that the (0, 0) mode for forward illumination takes the same value as that of backward illumination, inferring that (0, 0) mode has no effect on broadband ALT effect. The diffraction efficiency at other diffraction orders except (0, 0) for forward illumination is a bit larger than that for backward illumination, which unambiguously demonstrate the existence of ALT effect. So far, we get that the combined effects of the different number of diffraction orders and different diffraction efficiency at each order except (0, 0) contribute to the emergence of broadband ALT effect. What’s more, the cut-off point at 600 nm in Fig. 2(c) is related to Wood-Rayleigh anomaly wavelength given by $\lambda =\sqrt{\varepsilon }P$, where $\varepsilon$ is the permittivity in diffraction space (i.e. $\varepsilon =1$ in air for backward illulmination). For forward illumination, the large permittivity $\varepsilon =2.5281$ leads to the existence of a cut-off point at about 1500 nm, which is beyond our simulation range from 375 to 825 nm in Fig. 2(b).

**Figure 3:**
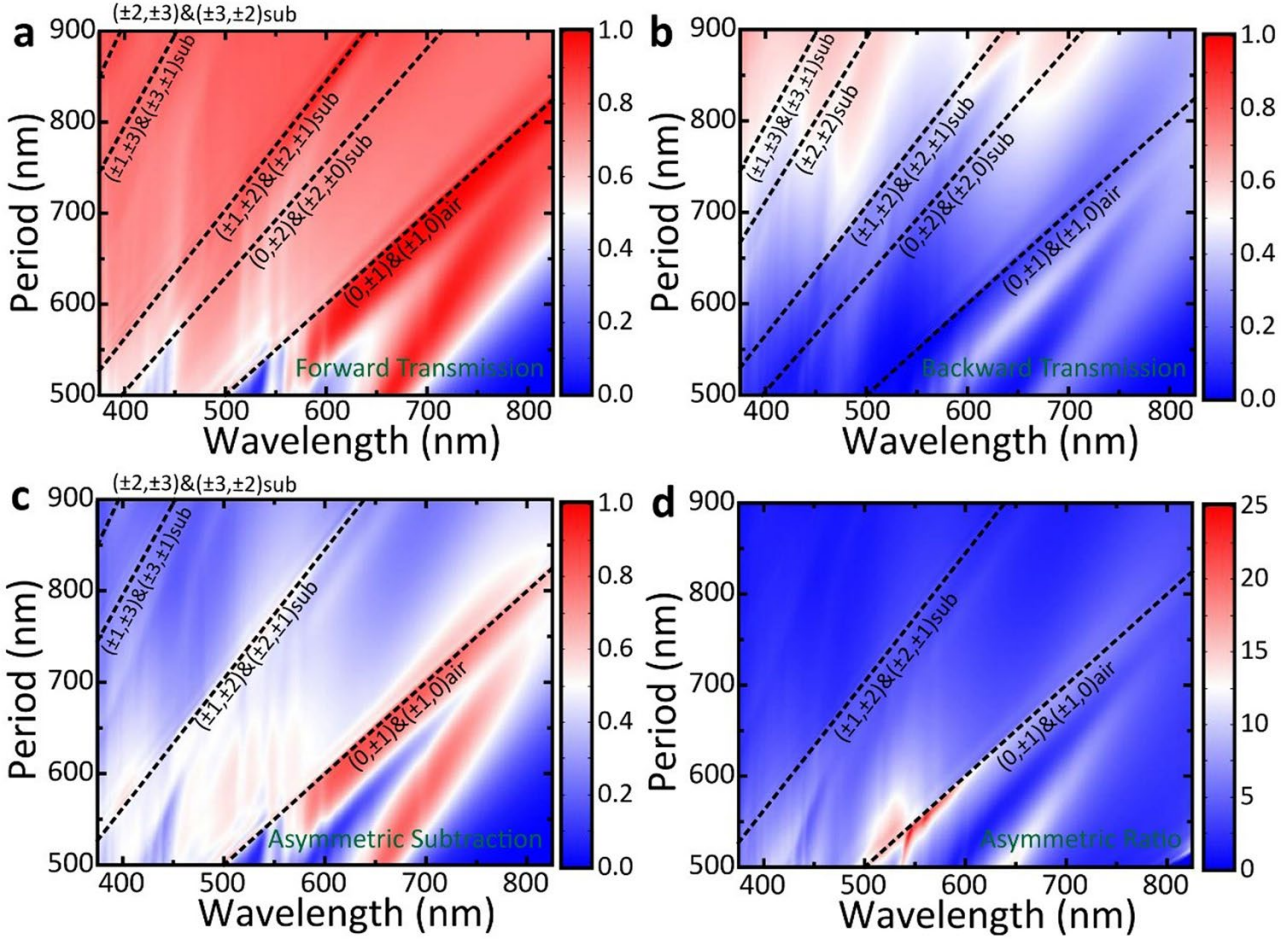
(**a**) and (**b**) Simulated forward (**a**) and backward (**b**) transmittance spectra versus lattice period *P* for normal incidence; (**c**) and (**d**) The calculated asymmetric subtraction and ratio based on (**a**) and (**b**). The inset dashed line is the diffraction orders (*m*, *n*) calculated by Eq. (6). The label ‘air’ and ‘substrate’ denote the related diffraction orders in air and substrate respectively. The detail structural parameters of MDCG used here are the same with that in Figs 1 and 2 except period *P*, where *h*_1_ = 75 nm, *h*_2_ = 480 nm, *r* = 180 nm, and ${n}_{l}$ = 1.59.

To gain more knowledge about the relationship between broadband ALT effect and diffraction effect, we investigate the transmittance of MDCG at various lattice periods. Figure 3(a) and (b) show the forward and backward transmittance spectra of MDCG as a function of lattice period *P*. The saltation lines represent the results of diffraction effect coincide well with theory as noted by the inset dashed lines. We can clearly see that forward transmittance is larger than backward transmittance, especially for diffraction order (0, ±1) and (±1, 0) in air. The calculated asymmetric subtraction and ratio as a function of wavelength $\lambda$ and lattice period *P* are shown in Fig. 3(c) and (d). Obvious ALT effect can be observed at the diffraction orders of (±1, 0) and (0, ±1) in air and (±1, ±2) and (±2, ±1) in substrate. We would like to emphasize that for the diffraction orders of (±1, 0) and (0, ±1) in air, the asymmetric ratio can even reach to 25 dB when *P* = ~550 nm. This giant asymmetric ratio disappears rapidly with the increasing of lattice period *P*. We also observe another obvious ALT region in the waveband of 550 nm to 800 nm, which is related to LSPR effect excited by Ag cylinder and will be discussed in detail later.

### LSPR effect in MDCG

**Figure 4:**
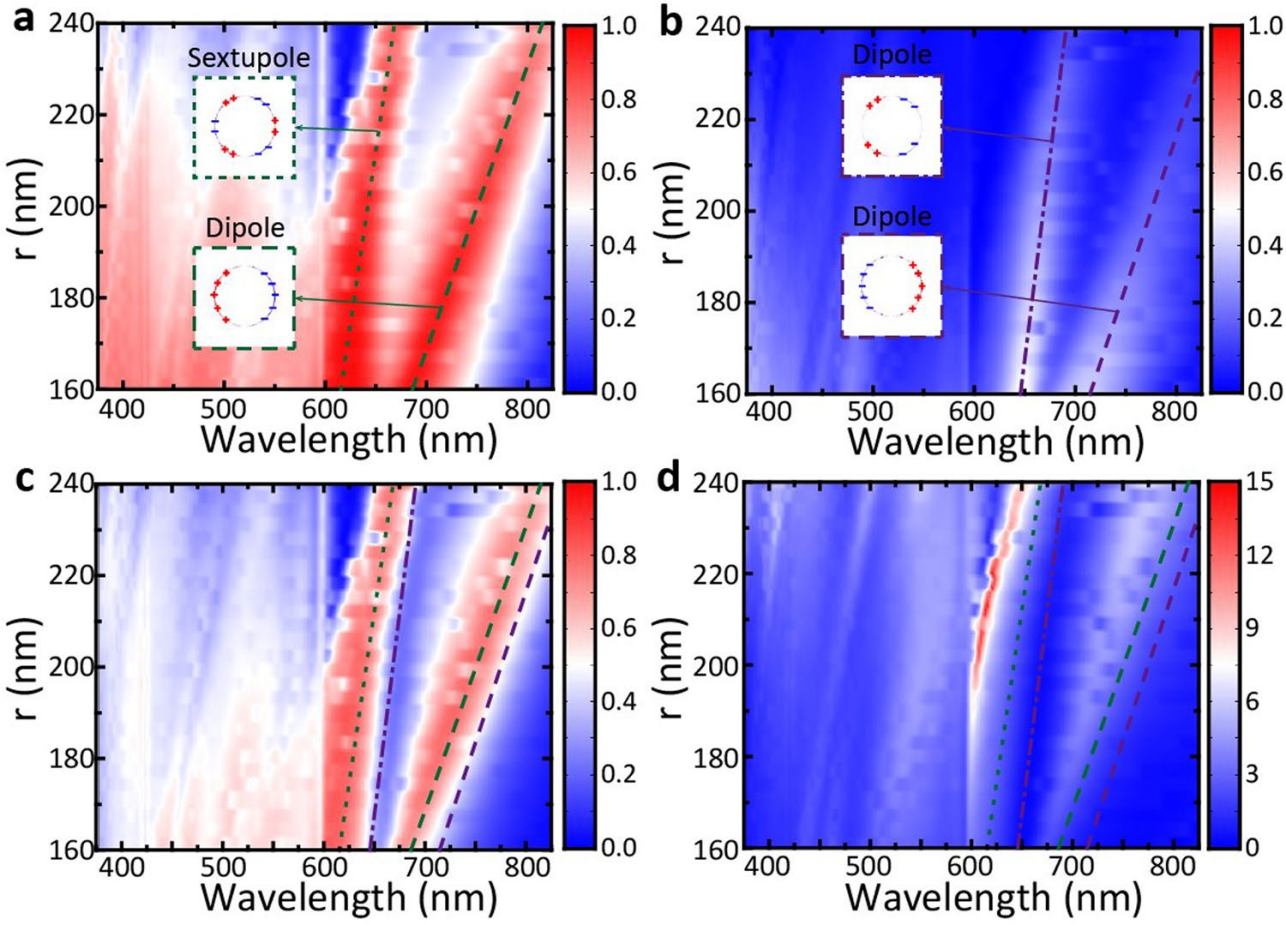
(**a**) and (**b**) Simulated forward (**a**) and backward (**b**) transmittance spectra versus the radius *r* of cylinder; (**c**) and (**d**) The calculated asymmetric subtraction and ratio based on (**a**) and (**b**). The doted, dashed and dash-dot lines denote the sextupole and dipole LSPR peaks, and the green and purple refer to forward and backward illumination, respectively. The inset images show the related charge distribution at these LSPR peaks in Ag cylinder. The refractive index of dielectric cylinder and substrate is ${n}_{l}$ = 1.59. The structural parameters are the same with Fig. 1, where *h*_1_ = 75 nm, *h*_2_ = 480 nm, and *P* = 600 nm.

LSPR, the collective electron oscillations in limited space, can be excited in Ag cylinder, which can affect diffraction efficiency, thus influencing the ALT effect. To analyze the influence of LSPR effect, we firstly investigate the transmittance of MDCG at various cylinder radius *r* as shown in Fig. 4. In fact, when fixing the values of lattice period *P* and refractive index ${n}_{l}$ of dielectric cylinder, the total number of diffraction orders, the related diffraction angles and the critical wavelength ${\lambda }_{c}$ will be uniquely identified according to Eqs (3) and (4). As a result, the two transmission peaks located in the range from 600 to 800 nm should be undoubtedly attributed to LSPR effect, which increase with the enlarging of Ag cylinder radius as shown in Fig. 4(a) and (b). For forward transmission, the LSPR peaks locate at 625 nm and 717 nm when the cylinder radius is 180 nm, which should refer to sextupole and dipole resonance, as indicated by the inset images; while for backward transmission, the LSPR peaks locate at larger wavelength, i.e. 659 nm and 745 nm. The different position of LSPR peaks of forward and backward transmittance, together with the transmission intensity difference, resulting in the large ALT effect around LSPR peaks (Fig. 4(c) and (d)). Especially for the sextupole dipole resonance, an asymmetric ratio of larger than 30 dB is observed in the wave range from 600 to 650 nm (Fig. 4(d)). It should be noted that the LSPR peaks at 625 nm and 717 nm for forward illumination are indeed the peaks of diffraction efficiency at (±1, 0) diffraction orders which are shown in Fig. 2. This implies that for forward illumination, these two LSPR modes enlarge the diffraction efficiency at higher orders, which can be suppressed under backward illumination. When the cylinder radius *r* is smaller than 190 nm, due to the existence of Wood-Rayleigh anomaly, there are cut-off points at 600 nm for the asymmetric subtraction and ratio and a weak ALT effect for the wavelengths smaller than 600 nm.

**Figure 5:**
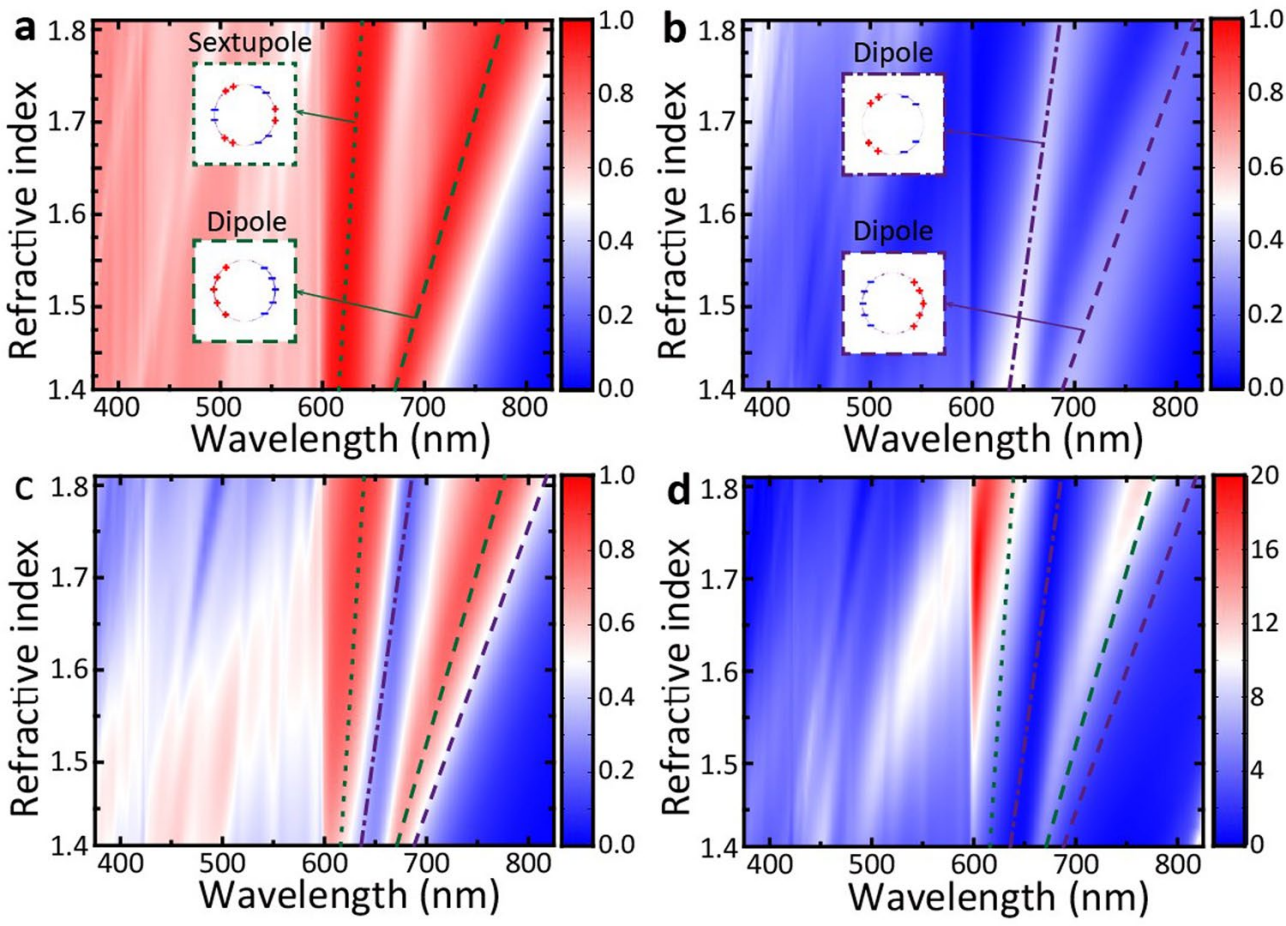
(**a**) and (**b**) Simulated forward (**a**) and backward (**b**) transmittance spectra versus ${n}_{l}$; (**c**) and (**d**) The calculated asymmetric subtraction and ratio based on (**a**) and (**b**). The doted, dashed and dash-dot lines denote the sextupole and dipole LSPR peaks, and the green and purple refer to forward and backward illumination, respectively. The inset images show the related charge distribution at these LSPR peaks in Ag cylinder. The radius of cylinder in (**c**) and (**d**) is 180 nm. The structural parameters are the same with Fig. 1, where *h*_1_ = 75 nm, *h*_2_ = 480 nm, and *P* = 600 nm.

The influence of refractive index *n* of the dielectric cylinder and substrate has also been investigated. As shown in Fig. 5, the transmittance and ALT effect of MDCG with increasing the index ${n}_{l}$ have a similar variation tendency with that of increasing the cylinder radius *r*. However, it should be underlined that the critical wavelength ${\lambda }_{c}$ at diffraction orders will change when increasing the refractive index ${n}_{l}$. In this case, the ALT effect located in the range from 600 to 800 nm in Fig. 5(c) and (d) should be ascribed to the combined effect of the sextupole LSPR and the diffraction at (1, 0) and (0, 1) orders in air. What’s more, one can expect that a higher performance of broadband ALT effect will be presented with the increasing of the refractive index ${n}_{l}$, which can be confirmed by Fig. 5(d) where we can see the asymmetric subtraction and ratio increase with enlarging the refractive index ${n}_{l}$.

### WGM in MDCG

**Figure 6:**
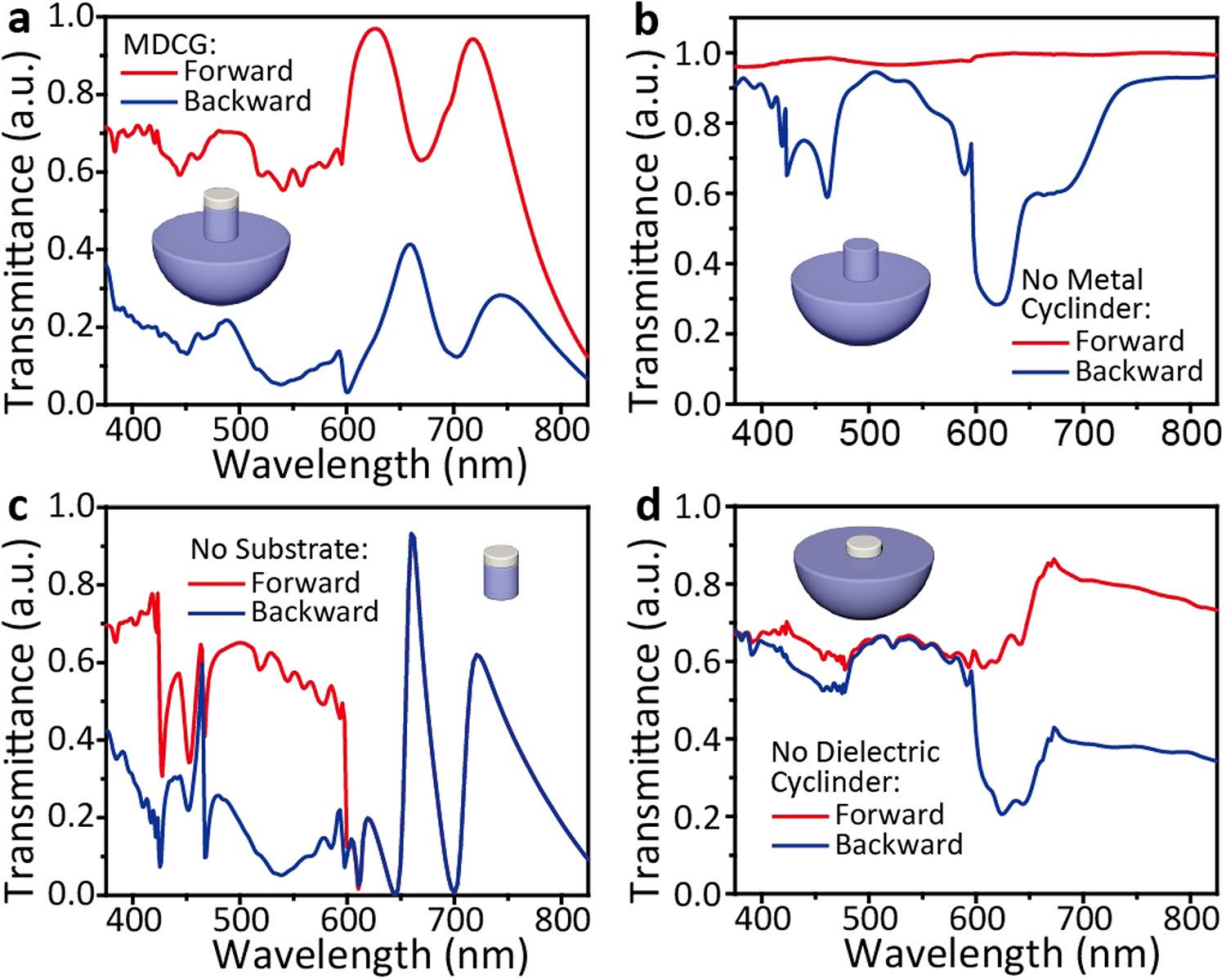
Forward (red curve) and backward (blue curve) transmittance spectra of MDCG (**a**), dielectric cylinder on dielectric substrate (**b**), Ag/dielectric cylinder (**c**) and Ag cylinder on dielectric substrate (**d**). The inset images are the related structure schematic diagram. The detail structural parameters used here is the same with that used in Fig. 1, where *h*_1_ = 75 nm, *h*_2_ = 480 nm, *r* = 180 nm, *P* = 600 nm, and ${n}_{l}$ = 1.59.

In addition to LSPR effect, the WGM excited in MDCG is another reason for the high-contrast broadband ALT effect. This WGM supported by the special microstructure of MDCG can affect the diffraction efficiency under forward and backward illumination. To analyze the structure-dependent WGM, we systematically investigate the contribution of each component of MDCG to ALT effect. Figure 6(a) shows the forward and backward transmittance spectra of the whole MDCG with optimal parameters, on which an obvious broadband ALT effect can be observed. When we place sole Ag cylinder or dielectric cylinder on the substrate, narrow-band ALT effects can be seen, which are shown in Fig. 6(b) and (d) (the detail diffraction information is shown in Figure S1 and S2). In addition, these ALT effects are mainly located in the waveband of larger than the Wood-Rayleigh anomaly wavelength (i.e. 600 nm), which can be mainly attributed to the influence of different refractive index between forward and backward transmission space. When we consider the Ag/dielectric cylinder structure, a well ALT effect is observed in the waveband smaller than the Wood-Rayleigh anomaly wavelength. Due to the same refractive index in forward and backward transmission spaces, the total number of diffraction orders and angles should keep the same as forward and backward illumination, which is confirmed by simulated results as shown in Figure S3. This well ALT effect should be closely related to the special structure, which can support WGM and enlarge the difference of diffraction efficiency between forward and backward illumination. The WGM, along with LSPR effect and difference of transmission spaces, results in the appearance of high-contrast broadband ALT effect.

**Figure 7:**
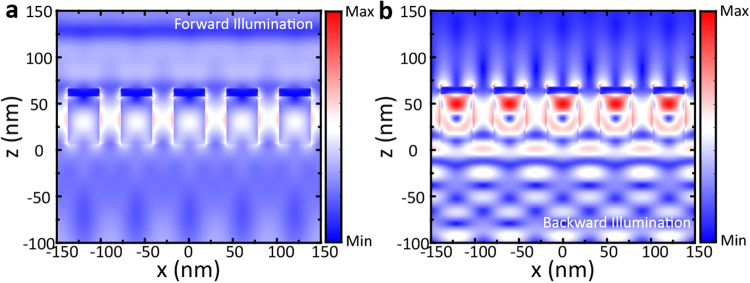
The profiles of electric field ${|E/{E}_{0}|}^{2}$ distribution for (**a**) forward illumination and (**b**) backward illumination at the wavelength of 615 nm. The detail structural parameters used here are the same with that used in Fig. 1, where *h*_1_ = 75 nm, *h*_2_ = 480 nm, *r* = 180 nm, *P* = 600 nm, and ${n}_{l}$ = 1.59.

To reveal the underlying mechanism of WGM, we investigate the electric field distribution for both forward and backward illuminations. Figure 7 shows the electric field distribution of MDCG at the peak (i.e. 615 nm, as shown in Fig. 1(d)) of asymmetric ratio between forward and backward illuminations. LSPR is excited between the slits of the cylinders. For forward illumination, the constructive interference of both SPP waves on Ag cylinder walls enhances the radiation transmission, causing an increasement of forward transmittance; while for backward illumination, most of the incident light is reflected to form resonant WGM in dielectric cylinder, where high electric intensity distribution is observed. Meanwhile, the reflected light interferes destructively with incident light, further suppressing radiation into the air. Thus, only a small part of optical energy can go through MDCG. In addition, LSPR at the corners of Ag cylinder for backward illumination is relatively weak, therefore the forward scattering and strong radiation modes cannot be excited.

### Diffraction Light transmission in grating-based ALT device substrate

**Figure 8:**
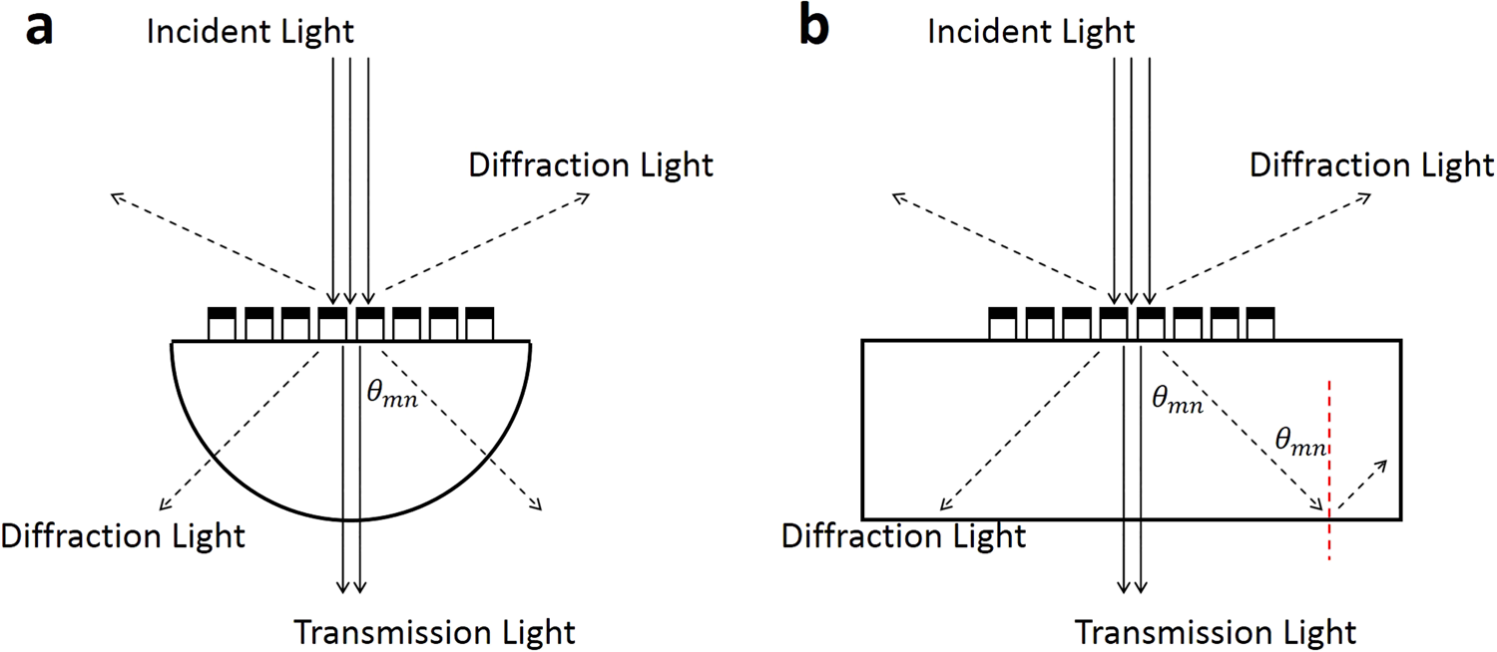
The schematics of our designed MDCG with different substrates: (**a**) semi-sphere substrate and (**b**) cuboid substrate. The size of substrate, i.e. the radius of the semi-sphere substrate or the length, width and height of the cuboid substrate, is large enough to avoid additional diffraction or interference effect.

Previous discussions are based on MDCG is placed on a semi-infinite substrate, which is reasonable for applications in fibers, solar cells and so on. But in most other cases, an independent ALT device is highly desired, where the diffraction lights should transmit out the substrate. However, the large diffraction angle in grating-based ALT devices may result in a total internal reflection at the interface between substrate and air for diffraction lights. To fully understand this phenomenon, we firstly calculate the critical angle in our case which is ${\theta }_{c}=arcsin(\frac{{n}_{l2}}{{n}_{l1}})=\arcsin (\frac{1}{1.59})\approx 43.3$ deg. The diffraction angle for (*m*, *n*) diffraction order can be easily calculated by Eq. (3), which is ${\theta }_{mn}=\arcsin (\frac{\lambda }{1.59})$ $\sqrt{{(\frac{m}{600})}^{2}+{(\frac{n}{600})}^{2}}$ in the optimized MDCG. When ${\theta }_{mn} > \,{\theta }_{c}$, we can get $\lambda  > \frac{600}{\sqrt{{m}^{2}+{n}^{2}}}$ nm, which means that all the diffraction lights with wavelengths larger than $\frac{600}{\sqrt{{m}^{2}+{n}^{2}}}$ nm can be reflected to the substrate for a grating placed on a cuboid substrate as shown in Fig. 8(b), which is a normal phenomenon for all the ALT devices based on the asymmetric diffraction effects. Certainly, if the wavelength of diffraction lights is smaller than $\frac{600}{\sqrt{{m}^{2}+{n}^{2}}}$ nm, the total internal reflection phenomenon will not happen. In order to guide the diffraction lights going out of the substrate, we employ a semi-sphere substrate as shown in Figs 1(a) and 8(a). In this case, the incident angles for diffraction lights at the sphere interface are nearly zero and all the diffraction lights can go out to the air again. In addition to the semi-sphere substrate, we can also employ other substrates to overcome this problem, such as gradient refractive index substrate.

## Conclusion

In this work, we propose and numerically demonstrate a high performance optical diode-like device based on MDCG placed on a semi-sphere substrate. The asymmetric subtraction and ratio can reach to about 0.5 and 4 dB in the waveband from 380 nm to 780 nm respectively, except for the small dip in the vicinity of 660 nm. The optimal asymmetric subtraction and ratio can reach to 0.8 and 13 dB. This excellent ALT effect should be attributed to special asymmetric diffraction effect, LSPR effect together with WGM supported by MDCG. The differences between forward and backward transmission space, i.e. dielectric substrate and air, enlarge the diffraction differences, including both the total number of diffraction orders especially for the wavelength larger than Wood-Rayleigh anomaly wavelength in air and diffraction intensity of each mode. Meanwhile LSPR effect and WGM play a leading role on diffraction efficiency. The different position of LSPR peaks excited in Ag cylinder under forward and backward illumination leads to a well ALT effect at wavelength larger than Wood-Rayleigh anomaly wavelength in air. In fact, LSPR effect excited under forward illumination greatly enlarge the diffraction efficiency at (1, 0) and (0, 1) orders in substrate. The WGM formed for backward illumination effectively prevent light going to air and promote ALT effect especially at wavelength smaller than Wood-Rayleigh anomaly wavelength in the air. In addition, the semi-sphere substrate can effectively guide diffraction light going out the substrate, resulting a real ALT device. These theoretical analyses improve our understanding of the ALT effect produced by gratings. And the easy-processing MCDG with high ALT performance for both polarized and unpolarized light may find potential applications in unidirectional electromagnetic field.

## Methods

We use FDTD calculations to simulate the optical properties and near field enhancement. A plane wave with wavelengths from 375 nm to 825 nm is placed in reflection space to illuminate MDCG, and the transmission light is collected by a monitor placed in transmission space. A periodic boundary condition is used in X and Y directions, and a perfect matched layer is used in Z direction. The thickness of the dielectric substrate is assumed to be infinite in simulation while for fabrication we can use semi-spherical surface to impede potential total reflection for higher diffraction orders. The height of metal and dielectric cylinders *h*_1_ and *h*_2_, radius *r* of cylinders, refractive index ${n}_{l}$ take specific parameters in different figures. The optical constants of silver are taken from the previously measured values^38^. A uniform mesh size of 2 nm was used in x, y, and z directions to ensure accuracy of electric field simulations inside the metal cylinders. And all simulation results have been normalized to the incident light power.

## Electronic supplementary material

Supplementary Information
