# Supplementary material for: Broadband Asymmetric Light Transmission at Metal/Dielectric Composite Grating
Source: Sci Rep. 2018 Jan 17;8:999. doi: 10.1038/s41598-018-19329-7 (PMC5772635; doi:10.1038/s41598-018-19329-7)
Supplement: Supplementary file 1 — Supplementary Information [file 41598_2018_19329_MOESM1_ESM.doc]

Supporting Information

**Broadband Asymmetric Light Transmission at Metal/Dielectric Composite Grating**

*Rui Zhu^†,1^, Xuannan Wu ^†,1^,* *Yidong Hou^†,*^, Gaige Zheng^†^, Jianhua Zhu^†^, and Fuhua Gao^†^*


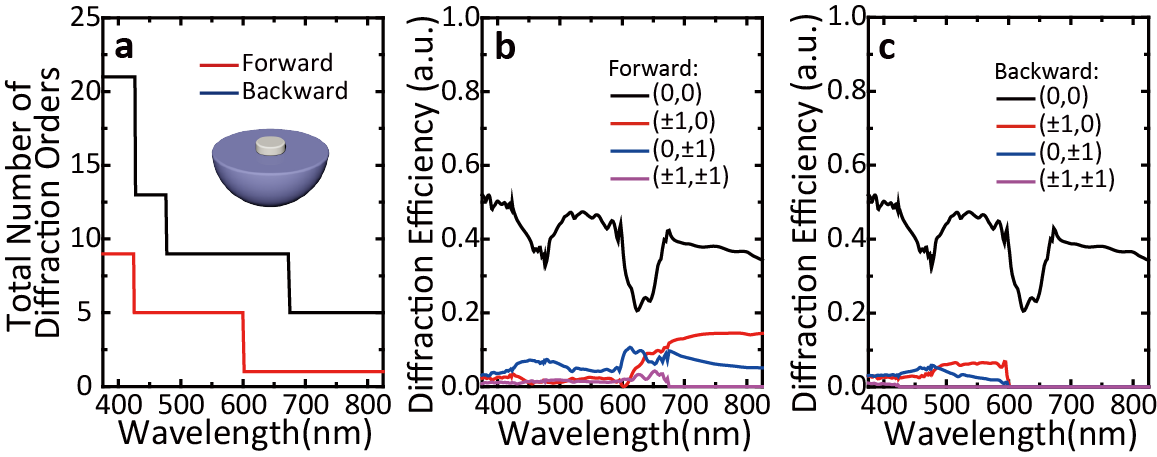
**Figure S1** Diffraction effect in periodic Ag cylinder grating on dielectric substrate. (a) Total number of diffraction orders as a function of wavelength in transmission space under forward (black curve) and backward (red curve) illumination. The inset image shows the related structure schematic diagram; (b) and (c) The diffraction efficiency at different diffraction orders under forward and back illumination respectively. The structural parameters used here are the same with that in Figure 1, where *h*_1_ =75 nm, *r* = 180 nm, *P* = 600 nm, and
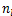
 = 1.59.


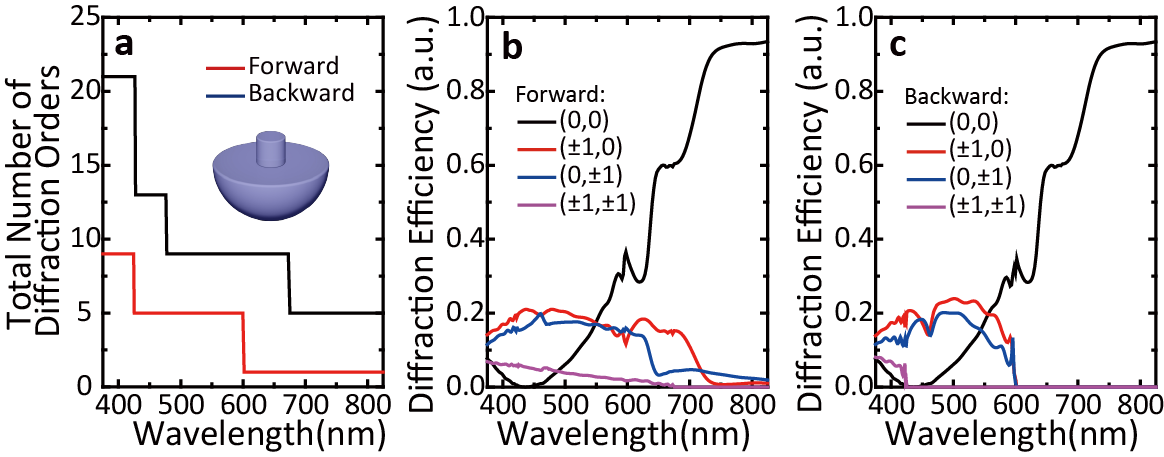
**Figure S2** Diffraction effect in periodic dielectric cylinder grating on dielectric substrate. (a) Total number of diffraction orders as a function of wavelength in transmission space under forward (black curve) and backward (red curve) illumination. The inset image shows the related structure schematic diagram; (b) and (c) The diffraction efficiency at different diffraction orders under forward and back illumination respectively. The structural parameters used here are the same with that in Figure 1, where *h*_2_ = 480 nm, *r* = 180 nm, *P* = 600 nm, and
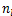
 = 1.59.


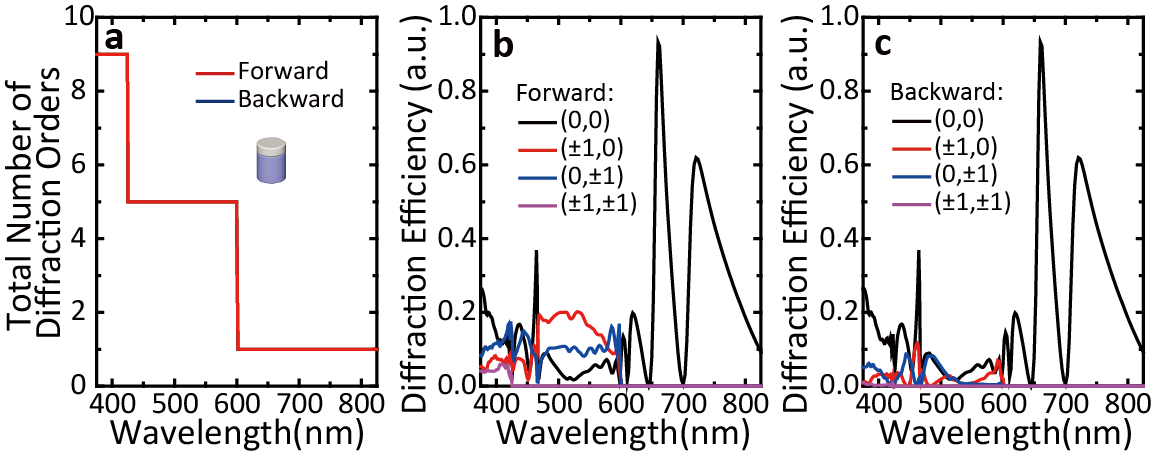
**Figure S3** Diffraction effect in periodic Ag/dielectric cylinder grating. (a) Total number of diffraction orders as a function of wavelength in transmission space under forward (black curve) and backward (red curve) illumination. The inset image shows the related structure schematic diagram; (b) and (c) The diffraction efficiency at different diffraction orders under forward and back illumination respectively. The structural parameters used here are the same with that in Figure 1, where *h*_1_ = 75 nm, *h*_2_ = 480 nm, *r* = 180 nm, *P* = 600 nm, and
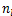
 = 1.59.


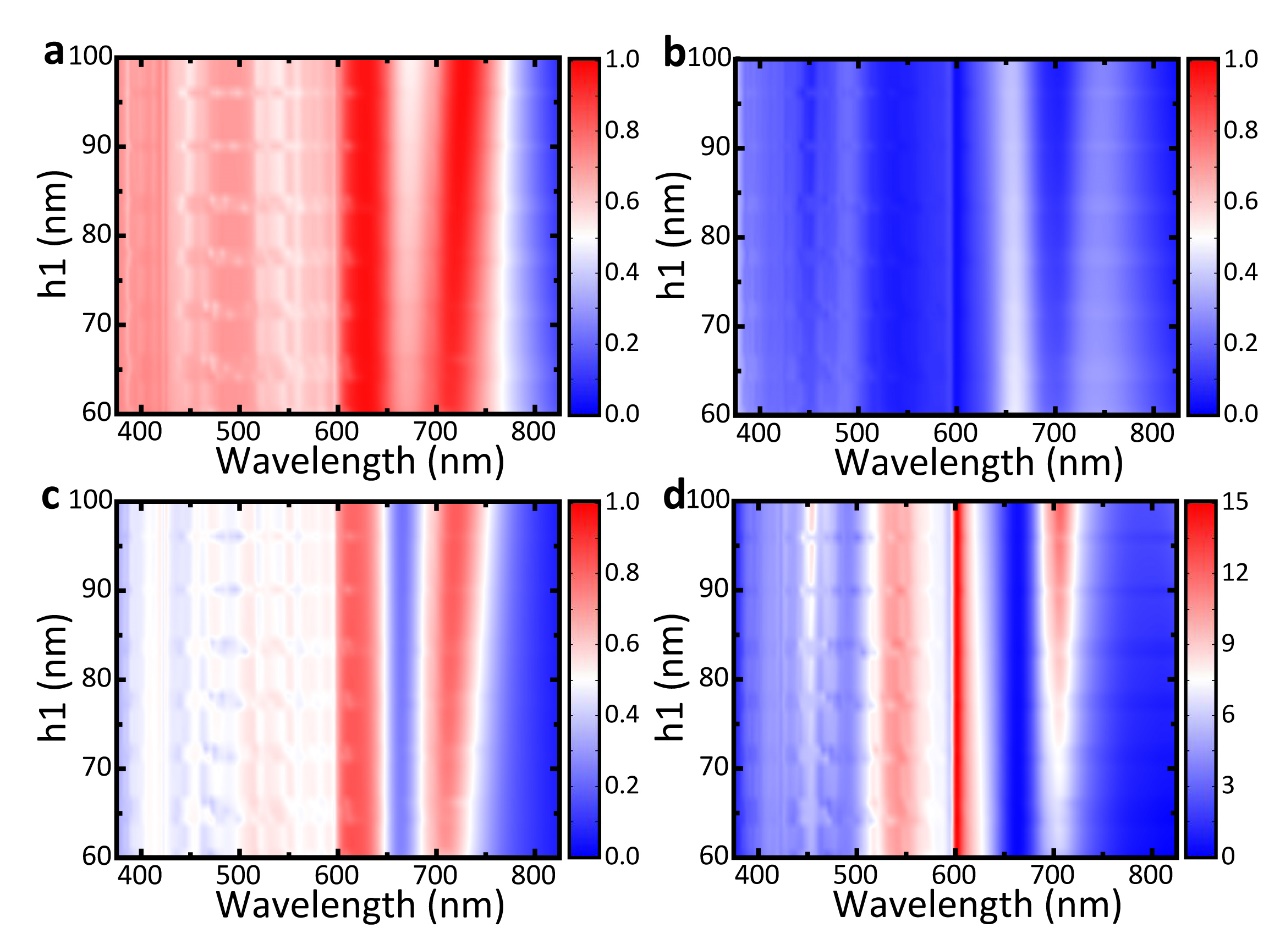


**Figure S4** (a) and (b) Simulated forward (a) and backward (b) transmittance spectra versus Ag cylinder height *h*_1_ for normal incidence; (c) and (d) The calculated asymmetric subtraction and ratio based on (a) and (b). The detail structural parameters of MCGS used here are the same with that in Figure 1 except cylinder height *h*_1_, where *h*_2_ = 480 nm, *P* = 600 nm, *r* = 180 nm, and
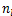
 = 1.59.


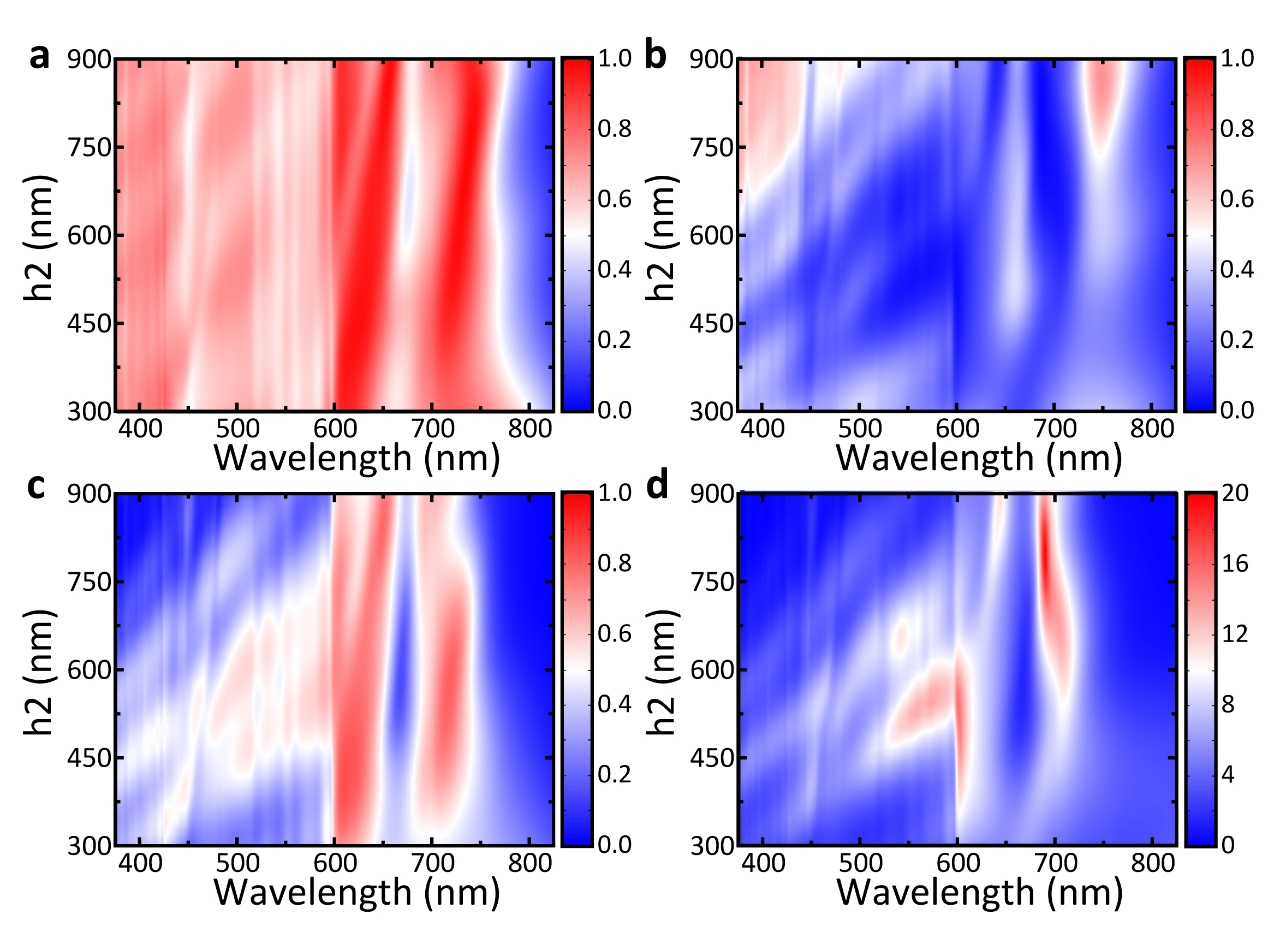


**Figure S5.** (a) and (b) Simulated forward (a) and backward (b) transmittance spectra versus dielectric cylinder height *h*_2_ for normal incidence; (c) and (d) The calculated asymmetric subtraction and ratio based on (a) and (b). The detail structural parameters of MCGS used here are the same with that in Figure 1 except cylinder height *h*_2_, where *h*_1_ = 75 nm, *P* = 600 nm, *r* = 180 nm, and
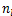
 = 1.59.
